# Enhanced Photocatalytic Fuel Denitrification over TiO_2_/α-Fe_2_O_3_ Nanocomposites under Visible Light Irradiation

**DOI:** 10.1038/s41598-017-08439-3

**Published:** 2017-08-10

**Authors:** Renkun Huang, Ruowen Liang, Haimei Fan, Shaoming Ying, Ling Wu, Xuxu Wang, Guiyang Yan

**Affiliations:** 1 Department of chemistry, Fujian province university key laboratory of green energy and environment catalysis, Ningde Normal University, Ningde, 352100 P.R. China; 2 State key laboratory of photocatalysis on energy and environment, Fuzhou University, Fuzhou, 350002 P.R. China

## Abstract

With increasingly stringent environmental regulations, the removal of nitrogen-containing compounds (NCCs) from gasoline fuel has become a more and more important research subject. In this work, we have successfully synthesized TiO_2_/α-Fe_2_O_3_ heterogeneous photocatalysts with different mass ratios of TiO_2_ *vs*. α-Fe_2_O_3_. Taking photocatalytic denitrification of typical alkali NCCs, pyridine, in gasoline fuel under visible light irradiation (λ ≥ 420 nm) as the model reaction, the TiO_2_/α-Fe_2_O_3_ hybrids have exhibited enhanced photocatalytic activity compared with pure TiO_2_ and α-Fe_2_O_3_, giving a pyridine removal ratio of ∼100% after irradiation for 240 min. The improved photocatalytic performance can be attributed to the integrative effect of the enhanced light absorption intensity and more efficient separation of photogenerated electron-hole pairs. Importantly, this type of heterogeneous photocatalysts can be easily separate in the reaction medium by an external magnetic field that is very important for industrial purpose. In addition, major reaction intermediates have been identified by the liquid chromatograph-mass spectrometer (HPLC-MS) and a tentative photocatalytic denitrification mechanism has been proposed.

## Introduction

The nitrogen-containing compounds (NCCs) in gasoline fuel are one of the most alarming environmental concerns to date. Crude gasoline fuel naturally contains a high concentration of NCCs, such as pyridine, indoles, nitrides and their derivatives^1^. Once released into the atmospheric environment, their combustion products (e.g., NO_2_, NO and unburned hydrocarbon particles) will cause photochemical smog and resulting in serious hazardous effects on ecosystems and human health^1, 2^. The removal of nitrogen containing compounds from gasoline is currently achieved by catalytic hydrodenitrification, adsorptive denitrogenation, oxidative denitrogenation, and photocatalytic denitrogenation^3–8^. Among the above methods, photocatalytic is a promising technique since it achieves the one-pot removal of NCCs by utilizing sunlight^7, 8^.

Semiconductor titanium oxide (TiO_2_) has always been regarded as one of the most common photocatalyst for the treatment of NCCs, because of its physical and chemical stability, simple preparation, nontoxicity, low cost, and unique electronic and optical properties^9, 10^. Nevertheless, two main drawbacks should be tackled before TiO_2_ can meet the actual application in large-scale NCCs denitrogenation. First, due to its large band gap (3.2 eV), TiO_2_ does not absorb photons in the visible region of the electromagnetic spectrum, which significantly reduces its solar energy conversion efficiency. Even worse, as frequently reported, the low charge mobility in TiO_2_ contributed to higher recombination rate of photogenerated electrons and holes, thereby limiting the catalytic activity^11, 12^. Many attempts have been made to realize the actual applications of TiO_2_ photocatalysts, such as nanostructuring (nanofibers, hollow sphere)^13–15^ and coupling with other materials (CuS, graphene, noble metal)^16–20^. Actually, combining TiO_2_ with other semiconductors to construct heterostructures is considered as one of the best approaches to effectively improve its solar energy conversion and effectively accelerate the separation of photoexcited charge carriers^21^. Therefore, the exploration of efficient semiconductor-coupled TiO_2_ nanocomposites with highly visible-light photocatalytic denitrogenation performance has become an attractive area of investigation.

Based on its abundance, stability, nontoxic nature, and much smaller band gap (2.3 eV), iron oxide (α-Fe_2_O_3_) in particular is a promising candidate for the development of efficient solar photocatalysts^22–25^. However, α-Fe_2_O_3_ has one significant drawback: its photocatalytic performance is limited by the high recombination rate of the photogenerated charge carriers. The introduction of magnetic component α-Fe_2_O_3_ might not only offer some synergetic enhancement of the catalytic activity by forming the hybrid structure, but also prevent the agglomeration of the catalyst nanoparticles during recovery^26^. TiO_2_ could be one of the best surface catalysts due to its matched band position with α-Fe_2_O_3_. Thus, it is reasonable to believe that this heterostructure has the enormous potential to increase the separation and transfer efficiency of photongenerated charge carriers and meanwhile conquer the drawbacks of pure TiO_2_ and α-Fe_2_O_3_^27, 28^. Although there have been some reports on TiO_2_ or α-Fe_2_O_3_ photocatalysts. It should be noted that they always focus on the elimination of NO_x_ from the flue gas^10, 29, 30^. Especially, the utilization of TiO_2_/α-Fe_2_O_3_ composites in photocatalytic denitrification of NCCs from the original gasoline fuel has remained unavailable so far.

Herein, we report for the first time that the TiO_2_/α-Fe_2_O_3_ composites can be utilized as photoactive and durable photocatalysts toward the denitrification of one kind of typical NCCs, pyridine, in original gasoline fuel under ambient conditions. Alkali nitride, pyridine, has been chosen as the target of the research owing to its widely existing and greatly influence the quality of fuel oil. The results have demonstrated that, under the irradiation of visible light, the TiO_2_/α-Fe_2_O_3_ exhibit enhanced photocatalytic performance, compared to individual α-Fe_2_O_3_ at the same conditions. The origin accounting for the improved photoactivities and the underlying reaction mechanisms have been studied in terms of a series of characterization and trapping experiments. Moreover, the possible photocatalytic reaction mechanism has also been investigated in detail.

## Results

### Characterizations

**Figure 1:**
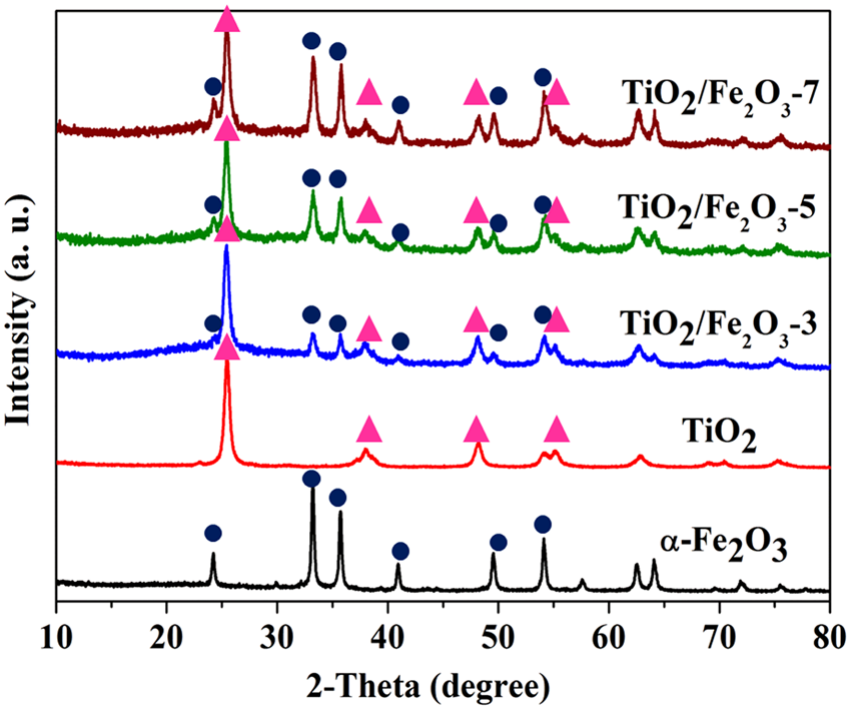
XRD patterns of pure TiO_2_, pure α-Fe_2_O_3_ and TiO_2_/Fe_2_O_3_ composites.

Figure 1 shows the XRD patterns of the as-prepared TiO_2_, Fe_2_O_3_ and TiO_2_/α-Fe_2_O_3_ composites (with 30, 50, and 70 wt.% of Fe_2_O_3_, designated as TiO_2_/Fe_2_O_3_-3, TiO_2_/Fe_2_O_3_-5, and TiO_2_/Fe_2_O_3_-7, respectively). It is obvious that the all the diffraction peaks of the TiO_2_ belong to pure anatase structure of TiO_2_ (JCPDS 21–1272). Meanwhile, six crystal peaks at 2θ = 24.1°, 33.2°, 35.6°, 40.8°, 49.5°, and 54.1°can be indexed as (012), (104), (110), (113), (024) and (116) reflection of Fe_2_O_3_ (α-Fe_2_O_3_, JCPDS 89–8103)^28^. For the TiO_2_/Fe_2_O_3_ composites with different mass ratios, all the reflection peaks could be indexed to hematite phases of Fe_2_O_3_ and anatase phases of TiO_2_, indicating the successfully combined anatase TiO_2_ with α-Fe_2_O_3_ in the composites. No peak from impurity phase was found, indicating the high purity of the as-prepared composites. It is easy to observe that the main characteristic diffraction peaks of the TiO_2_/Fe_2_O_3_ composites did not noticeably change after TiO_2_ hybridized with α-Fe_2_O_3_, suggesting that the calcination process could not destroy the crystal of TiO_2_.

**Figure 2:**
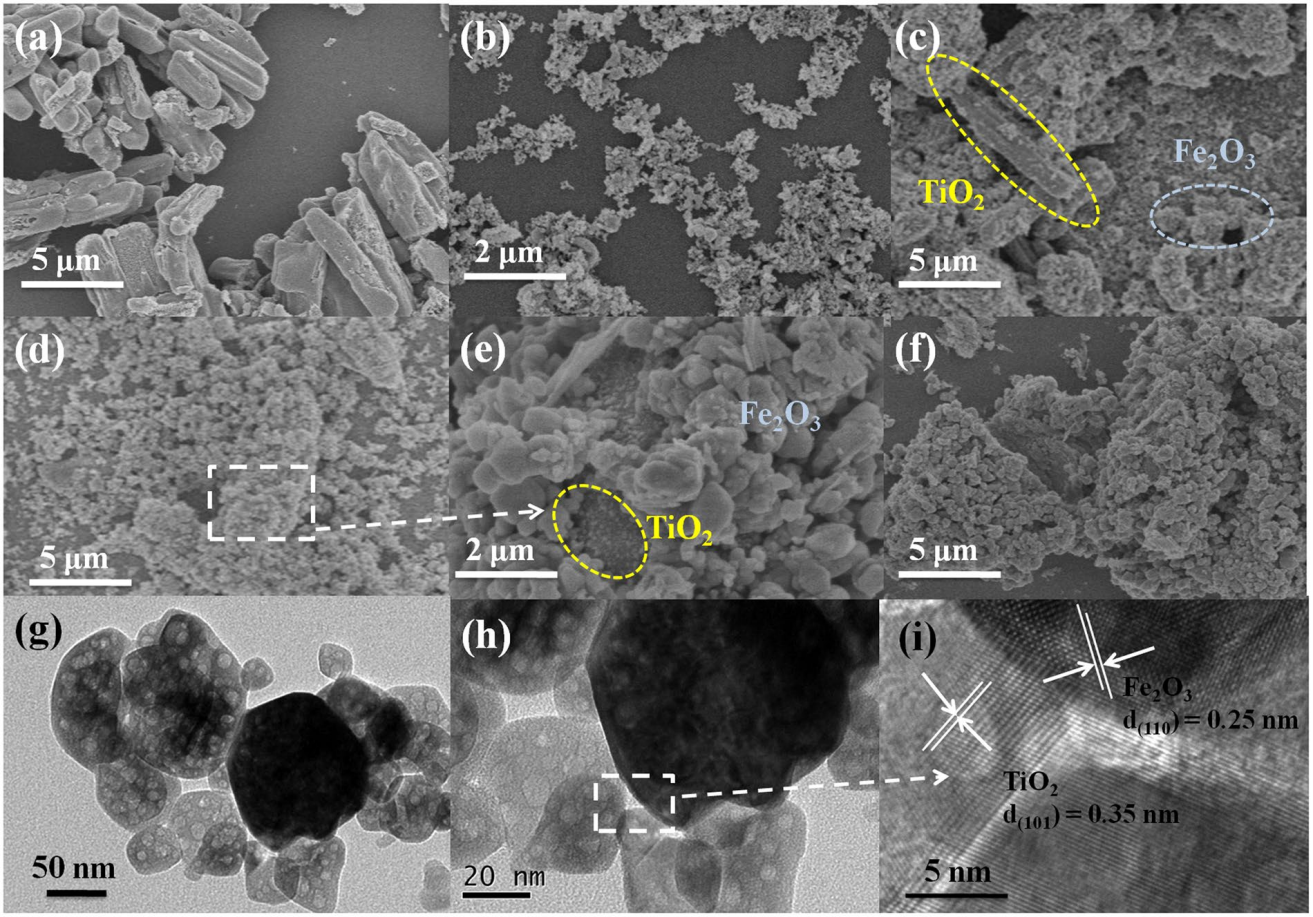
SEM images of (**a**) TiO_2_, (**b**) α-Fe_2_O_3_, (**c**) TiO_2_/Fe_2_O_3_-3; (**d**,**e**) TiO_2_/Fe_2_O_3_-5 (**f**) TiO_2_/Fe_2_O_3_-7, and (**g**,**h**) TEM images of as-prepared TiO_2_/Fe_2_O_3_-5; (**i**) HRTEM images of the TiO_2_/Fe_2_O_3_-5.

The surface morphology and microstructure information of the as-synthesized samples have been characterized by SEM and TEM. As seen in Fig. 2(a), the as-prepared TiO_2_ exhibit the 1D morphology with an average length of *ca*. 5–7 μm and an average diameter of *ca*. 50–100 nm, which is consistent with the previous report^31^. After the deposition of α-Fe_2_O_3_ onto the TiO_2_ microrods, Fe_2_O_3_ particles are densely coated onto the surface of the TiO_2_ microrods to form a hetero-interface between TiO_2_ microrods and Fe_2_O_3_ particles (Fig. 2(b–f)). It can be also found that, the quantity of α-Fe_2_O_3_ coated on the surface of the TiO_2_ increases gradually along with the enhancement of the loading amount of α-Fe_2_O_3_. Simultaneously, it could be observed from the TEM images (Fig. 2(g and h)), α-Fe_2_O_3_ particles are tightly combined with TiO_2_ microrods via a simple calcination process. This is further confirmed from a representative HRTEM image of the TiO_2_/Fe_2_O_3_-5 composite. From the Fig. 2(i), we could clearly observe two different crystal lattices corresponding to anatase-TiO_2_ (d_101_ = 0.35 nm) and α-Fe_2_O_3_ (d_110_ = 0.25 nm). The EDS elemental mapping also confirms that Fe, Ti and O elements are uniformly distributed in the TiO_2_/Fe_2_O_3_-5 (Fig. S1). All these results gave solid evidence that TiO_2_ microrods and α-Fe_2_O_3_ were successfully coupled together to form TiO_2_/Fe_2_O_3_ photocatalysts.

**Figure 3:**
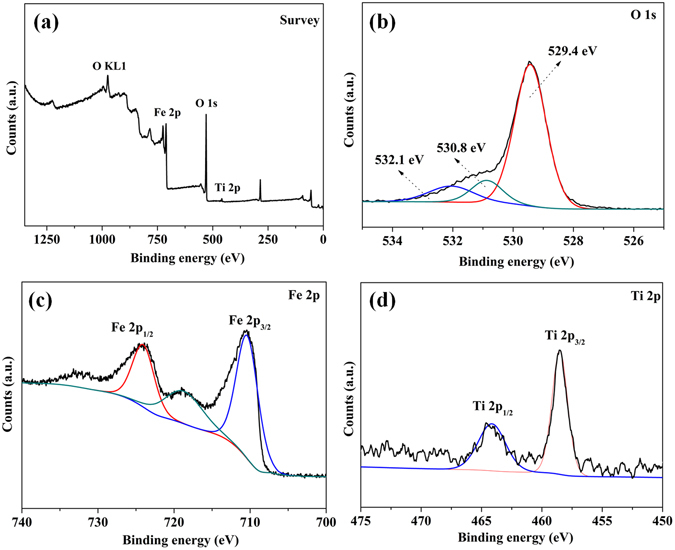
XPS patterns of TiO_2_/Fe_2_O_3_-5.

X-ray photoelectron spectroscopy (XPS) is performed to study the structural and chemical state of the elements present in TiO_2_/Fe_2_O_3_ nanocomposites. As shown in Fig. 3(a), the survey spectrum of the TiO_2_/Fe_2_O_3_-5 shows the pronounced featured signal of O 1 s, Fe 2p and Ti 2p, indicating that the Fe_2_O_3_ particles are successfully immobilized on the surface of the TiO_2_. The weak signal of Ti is maybe due to the covering effect of Fe_2_O_3_ in the TiO_2_/Fe_2_O_3_-5. Figure 3(b) shows the high-resolution XPS spectrum of the O1s. Beside the component of O1s lattice centered at 529.4 eV, two bands at 530.8 eV and 532.1 eV are detected and attributed to the presence of coordinatively unsaturated oxygen species (surface defects)^32^, which belong to the lattice oxygen combined with Fe^3+^ and Ti^4+^. Figure 3(c) shows the high-resolution XPS spectra of Fe 2p. The binding energies of 710.3 and 724.2 eV with a satellite signal at 718.9 eV are characteristic of Fe(III) in Fe_2_O_3_, which is due to spin-orbit splitting. The peak separation, namely, Δ = 2p_1/2_–2p_3/2_ = 13.9 eV, which is very similar to those reported for α-Fe_2_O_3_^33^. Figure 3(d) shows peaks at 458.5 and 464.1 eV and are assigned to Ti 2p_3/2_ and Ti 2p_1/2_ core levels. The difference between the Ti 2p core levels is 5.6 eV, indicating the normal state of Ti^4+^ in the anatase TiO_2_^34, 35^. The shoulder of the Ti 2p_3/2_ peak corresponds to a band at 458.5 eV. This band is assigned to formation of a Ti-O-Fe bond in the interface of TiO_2_/Fe_2_O_3_, which indicated the formation of TiO_2_/Fe_2_O_3_. Similar observation was also reported for TiO_2_/Fe_2_O_3_ coatings by Zhang and Lei^36^. The amount of Fe_2_O_3_ deposited on TiO_2_/Fe_2_O_3_ has been determined by ICP. It is found that the loading percentage of Fe_2_O_3_ in samples of TiO_2_/Fe_2_O_3_-3, TiO_2_/Fe_2_O_3_-5 and TiO_2_/Fe_2_O_3_-7 are 27.1%, 45.6%, 66.0%, respectively. It is also demonstrated that the calcination approach is an effective technique of immobilizing Fe_2_O_3_ onto the TiO_2_, because the content of Fe_2_O_3_ only slightly less than the theoretical content. For further information of the surface acidity of TiO_2_/Fe_2_O_3_ composites, the temperature-programmed desorption (TPD) of ammonia has been carried out. As shown in Fig. S2, the peaks at 560 °C can be assigned to desorption of ammonia from Lewis acid sites, which is due to the presence of unsaturated surface Ti^4+^ ions^37^. Moreover, The appearance of peak at 275 °C in NH_3_-TPD curve of TiO_2_/Fe_2_O_3_-5 can be associated with ammonia desorption from surface Fe^3+^, which play role of medium Lewis acid centers^38^. The NH_3_-TPD measurements results have confirmed the strong surface acidity of TiO_2_/Fe_2_O_3_ composites. Thus, the Lewis acid surface of TiO_2_/Fe_2_O_3_ is benefit to absorbing Lewis base, pyridine to obtain a better photocatalytic activity.

**Figure 4:**
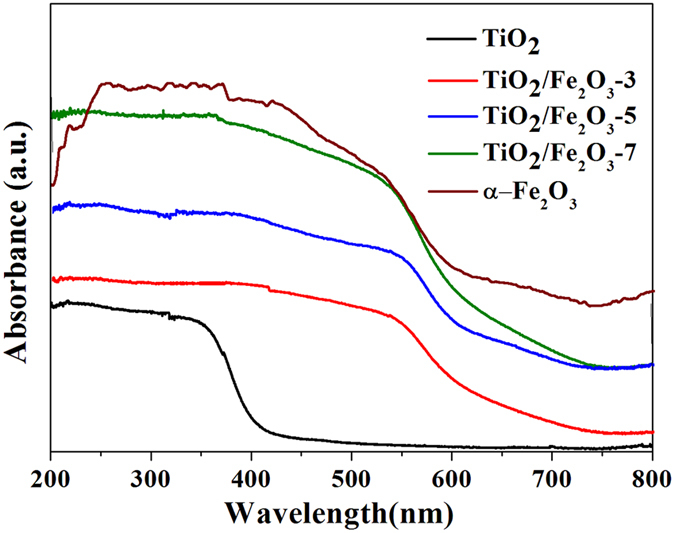
UV-vis absorption spectra of TiO_2_, pure α-Fe_2_O_3_ and TiO_2_/Fe_2_O_3_ composites.

The optical properties of the as-synthesized pure TiO_2_, α-Fe_2_O_3_ and TiO_2_/Fe_2_O_3_ composites have been characterized by UV-vis diffuse reflectance spectroscopy (DRS) and the results are shown in Fig. 4. TiO_2_ shows a typical absorption band edge at 390 nm, which originated from its band gap of 3.18 eV and in accordance with the reported value in the literatures^39^. As for the α-Fe_2_O_3_, it exhibits strong light absorption over the visible range, even extending to the infrared region, which is caused by 2(^6^A_1_) → (^4^T_1_) ligand field transition of Fe^3+^. With the integration of Fe_2_O_3_, the optical absorption of the composites in the visible light region is greatly improved, which is in accordance with the color change of the samples from white to reddish brown. Therefore, the enhanced absorbance of light is expected to improve the visible-light-driven photocatalytic activity for a target reaction. This inference is well verified by the photocatalytic testing of TiO_2_/Fe_2_O_3_ composites toward denitrogenation of NCCs under visible light irradiation.

### Photocatalytic properties

**Figure 5:**
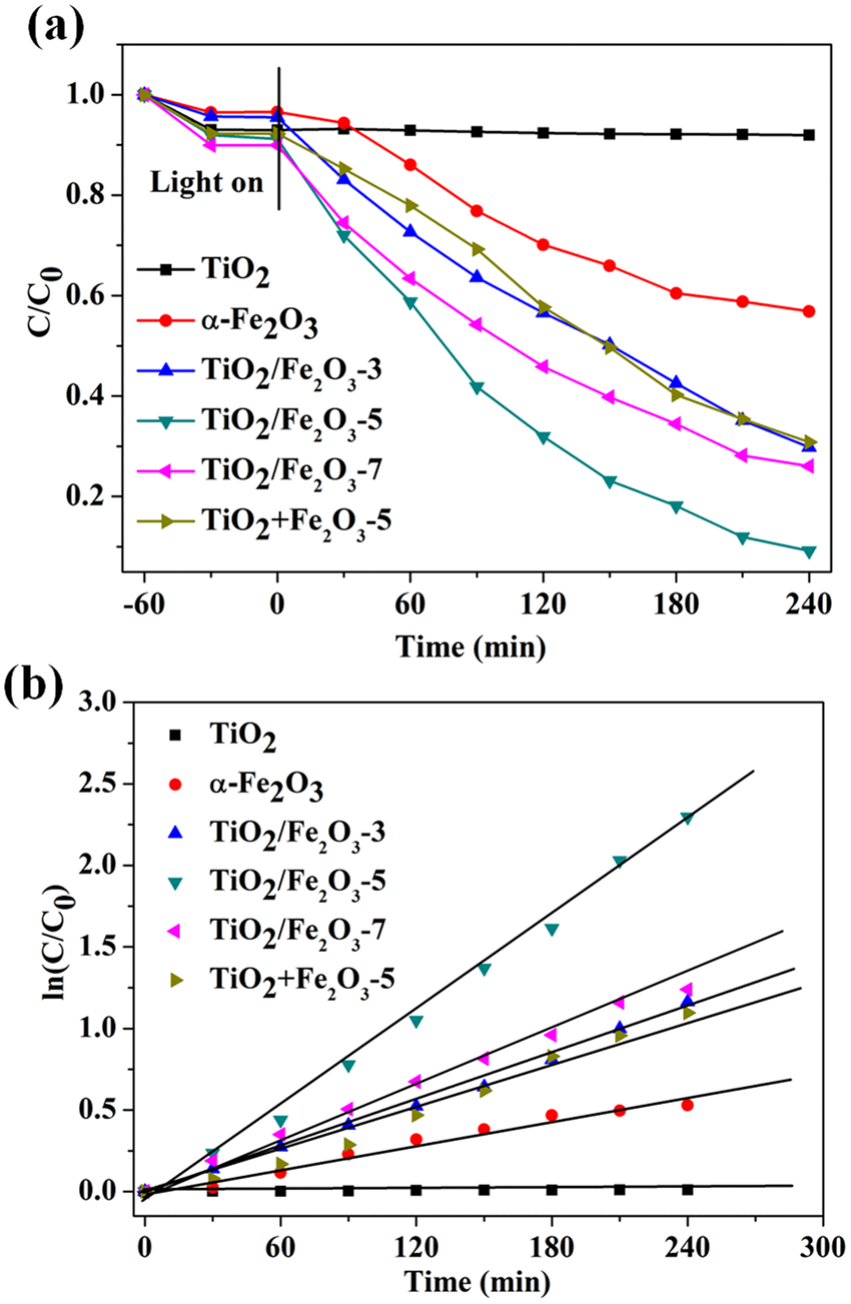
(**a**) Photocatalytic denitrogenation of pyridine over TiO_2_, α-Fe_2_O_3_ and TiO_2_/Fe_2_O_3_ composites with different mass ratios of Fe_2_O_3_; (**b**) The pseudo-first order rate constants of pyridine photocatalytic denitrogenation over TiO_2_, α-Fe_2_O_3_ and TiO_2_/Fe_2_O_3_ with different mass ratios of Fe_2_O_3_. Reaction conditions: 40 mg of photocatalyst, 40 mL of 100 μg/g pyridine.

The photocatalytic activities of TiO_2_/α-Fe_2_O_3_ composites have been evaluated by the photocatalytic denitrogenation of pyridine under visible light irradiation (λ ≥ 420 nm). Blank experiments have been first carried out to demonstrate the photocatalytic nature of the reaction (Fig. S3). Apparently, the denitrogenation of pyridine hardly occurs in the absence of photocatalyst or light. Instead, the denitrogenation of pyridine proceeds smoothly in the presence of photocatalyst. Importantly, TiO_2_/Fe_2_O_3_-5 composites exhibit much higher photocatalytic activity than that of TiO_2_ (~0%) and α-Fe_2_O_3_ (44%) under identical experimental conditions, respectively. The reduction ratio is rapidly increased to ~100% after visible light irradiation (λ ≥ 420 nm) for 240 min. Moreover, such photoactivity is higher than that of TiO_2_ + α-Fe_2_O_3_, which is prepared by simply mixing TiO_2_ and α-Fe_2_O_3_ in proper proportions under identical conditions (Fig. 5(a)). Fig. 5(b) is the photocatalytic reaction kinetics of pyridine photocatalytic denitrogenation in octane based on the data plotted in Fig. 5(a). As can be observed, photocatalytic denitrogenation of pyridine approximately follows pseudo-first-order kinetics, as evidences by the linear plot of ln(C_0_/C_t_) *vs*. reaction time t. As displayed in Fig. 5(b), the TiO_2_/Fe_2_O_3_-5 composite has the highest rate constant (0.0097 min^−1^) among all of the samples. The kinetic rate constants follow the order TiO_2_/Fe_2_O_3_-5 (0.0097 min^−1^) > TiO_2_/Fe_2_O_3_-7 (0.0050 min^−1^) > TiO_2_/Fe_2_O_3_-3 (0.0047 min^−1^) > α-Fe_2_O_3_ (0.0024 min^−1^) > TiO_2_ (0.0001 min^−1^). Therefore, we can draw the conclusion that coating TiO_2_ microrods with an ultrathin α-Fe_2_O_3_ layer could lead to the obvious photoactivity enhancement toward denitrogenation reactions. The results indicate that the effective interfacial hybridization between TiO_2_ and α-Fe_2_O_3_ contributes to the remarkably enhanced photoactivity, thus making TiO_2_/Fe_2_O_3_ composites be an efficient photocatalyst for denitrogenation of NCCs.

Besides the excellent photo-denitrogenation efficiency, the stability and recyclability of photocatalysts is another significant factor in their practical application. To confirm the photostability of the as-prepared photocatalysts, the recycling tests for pyridine denitrogenation have been conducted with sample TiO_2_/Fe_2_O_3_-5. After each cycling experiment, the photocatalyst was separated from the aqueous suspension by filtration and washed with ethanol several times. And then, the photocatalyst was centrifuged at 4500 rpm for 5 min and dried in vacuum at 100 °C for 2 h. As shown in Fig. S4(a), the results of recycling tests indicate no significant loss of photocatalytic activities after four cycles, suggesting that the TiO_2_/Fe_2_O_3_-5 photocatalyst is stable during the photocatalytic reaction. XRD results reveal that no significant changes are observed in the crystal structure of TiO_2_/Fe_2_O_3_-5 before and after the catalytic reaction (Fig. S4(b)). Additionally, the separability of the TiO_2_/Fe_2_O_3_-5 magnetic composites has also been tested by placing a magnet near the glass bottle after dispersing the TiO_2_/Fe_2_O_3_-5 in octane (Fig. S4(c)). It is observed that the magnetic particles are attracted towards the magnet within 1 min. The magnetic properties of the resultant TiO_2_/Fe_2_O_3_ have been investigated at room temperature by vibrating sample magnetometry (VSM) in the field range from - 4 to + 4 KOe (Fig. S5). The samples at room temperature have been measured to be 8.3 emu/g^−1^, revealing strong magnetic properties. The curve presents a magnetic hysteresis loop, which also depicts the strong magnetic response to a varying magnetic field. The above results directly demonstrate the convenient separation of the TiO_2_/Fe_2_O_3_-5 from liquids using an external magnetic field.

## Discussion

In order to investigate the reasons for obvious photoactivity enhancement toward denitrogenation of pyridine over the photocatalysts, the surface area measurement and photoelectrochemical experiments have been performed. The Brunauer-Emmett-Teller (BET) surface areas of the as-prepared composites have been investigated using nitrogen adsorption-desorption experiments. As shown in Fig. S6, the isotherm for three samples exhibited a type IV with a H3 hysteresis loop according to the IUPAC classification. The BET surface areas of the pristine TiO_2_, and Fe_2_O_3_ have been calculated to be 19.5 and 33.8 m^2^/g, respectively. To clearly see the variations of original-TiO_2_ after α-Fe_2_O_3_ decoration, we summarize the BET surface areas of the samples in Table 1. The results show that after coupling with α-Fe_2_O_3_, the surface area of the composites revealed a slight increase compared with TiO_2_. However, for all of these samples, there are no line-relationship between the surface areas and the observed photoactivity order, that is, TiO_2_/Fe_2_O_3_-5 > TiO_2_/Fe_2_O_3_-7 > TiO_2_/Fe_2_O_3_-3 > α-Fe_2_O_3_ > TiO_2_, cannot be attributed to the difference of surface area.

**Table 1:** BET surface area, reaction rate constant and normalized rate constant of TiO_2_, α-Fe_2_O_3_ and TiO_2_/Fe_2_O_3_ composites.

Samples	BET (m^2^/g)	K [min^−1^]	K’[g.min^−1^. m^−2^, ×10^−5^]
TiO_2_	19.5	0.0001	0.51282
α-Fe_2_O_3_	33.8	0.0024	7.10059
TiO_2_/Fe_2_O_3_-3	24.0	0.0047	19.5831
TiO_2_/Fe_2_O_3_-5	27.1	0.0097	35.7934
TiO_2_/Fe_2_O_3_-7	29.5	0.0050	16.9492

**Figure 6:**
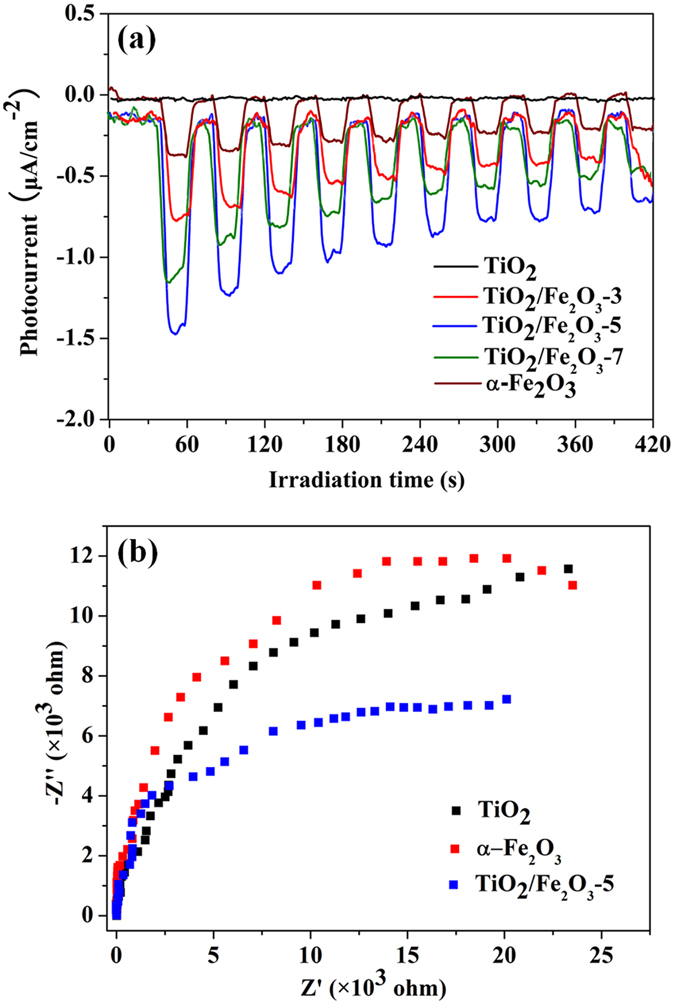
(**a**) Transient photocurrent response of TiO_2_, α-Fe_2_O_3_ and TiO_2_/Fe_2_O_3_ composites in 0.2 M Na_2_SO_4_ aqueous solution under irradiation of visible light (λ ≥ 420 nm); (**b**) Nyquist impedance plots of TiO_2_, α-Fe_2_O_3_ and TiO_2_/Fe_2_O_3_-5.

It is well established that TiO_2_ and Fe_2_O_3_ are provided with matchable energy band position, together with intimate interfacial contact confirmed by our TEM results, which is able to result in the efficient charge carriers transfer. This reference is verified by the electrochemical impedance spectra (EIS). It can be obviously seen from Nyquist impedance plots (Fig. 6(a)) that the TiO_2_/Fe_2_O_3_-5 shows depressed semicircles at high frequencies as compared to blank TiO_2_ and α-Fe_2_O_3_, suggesting that the charge-transfer resistance decreases. Therefore, a consensus is reached that the integration of TiO_2_ with α-Fe_2_O_3_ could improve the transfer of charge carriers, thereby efficiently hampering the recombination of electron-hole pairs^40^. The improved charge carrier separation and the prolonged lifetime of photogenerated electron-hole pairs can be confirmed by the photo-electrochemical experiments. As displayed in Fig. 6(b), the introduction of α-Fe_2_O_3_ enhances the photocurrent significantly, indicating a more efficient separation of the photoexcited electron-hole pairs.

A sequence of controlled experiments using different radical scavengers has been carried out in order to deeply understand the role of photogenerated radical species in the photocatalytic denitrogenation of pyridine over the photocatalysts. The results of adding different radical scavengers (ethanol for holes, CCl_4_ for electrons and isopropyl alcohol for OH·) over TiO_2_/α-Fe_2_O_3_ systems are shown in Fig. S7. When the holes scavenger, ethanol is added into the reaction system, the removal ratio of pyridine remarkably decrease, manifesting that the denitrogenation of pyridine is mainly related to photoinduced holes. Thus, the adsorbed pyridine in solution interacts with holes to form the corresponding radical cations, which further reacts with the trace H_2_O molecular and dioxygen, leading to the formation of CO_2_, H_2_O, NH_3_ and HCOOH (Fig. S8). For further information of the denitrogenation pathway of pyridine, the liquid chromatograph-mass spectrometer (HPLC-MS) has been carried out. As displayed in Fig. S9, the peak intensity m/z 81.5 is reduced after the photocatalytic fuel denitrification, meaning that the pyridine has been degraded successfully. Two new peaks appeared at m/z 85.0 and m/z 46.1 after the photocatalytic fuel denitrification, meaning that the pyridine has been translated into the protonated of intermediate products C_4_H_4_O_2_ and CH_3_NH_2_.

**Figure 7:**
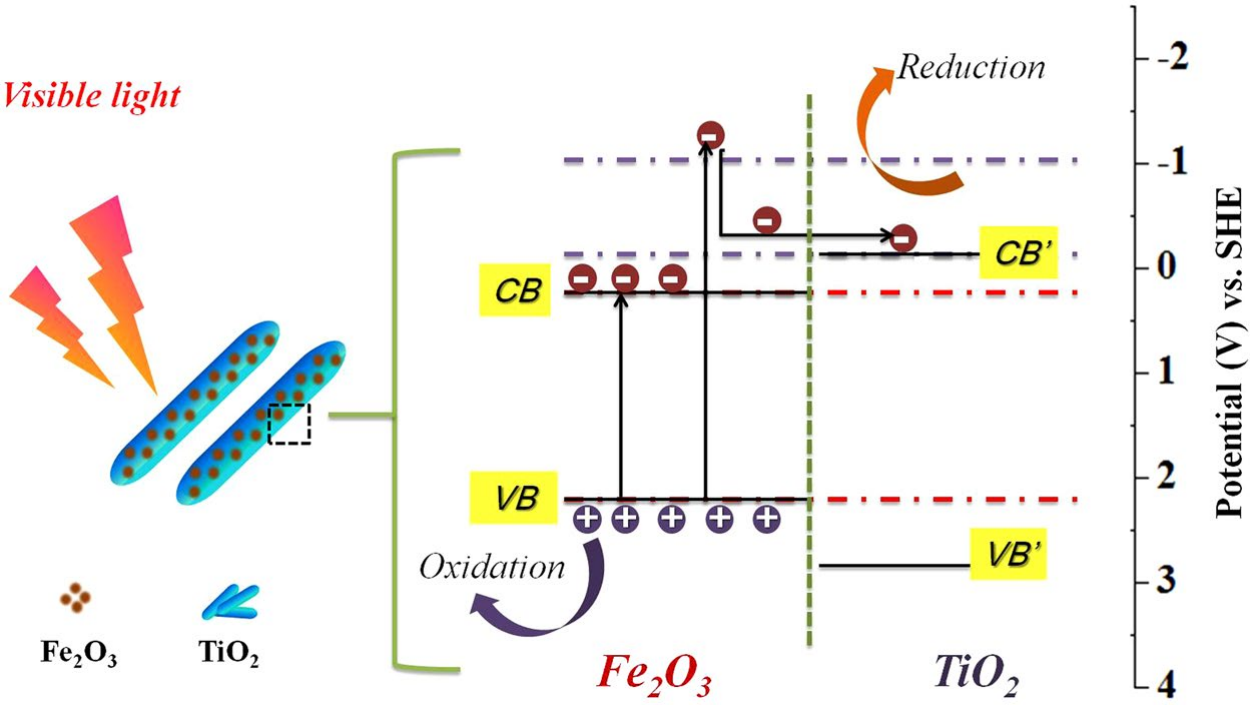
Possible mechanism of photocatalytic denitrogenation of pyridine over TiO_2_/Fe_2_O_3_-5.

Based on the discussion presented above and the experimental results, a synergistic photocatalytic mechanism of the TiO_2_/Fe_2_O_3_ catalyst was proposed, as illustrated in Fig. 7. It is clearly shown that the photogenerated electrons of α-Fe_2_O_3_ will be excited from valence band (VB) to its different energy-level conduction band (CB) position under the excitation of visible light, including high-energy region (−0.05 eV ~ −1.0 eV *vs*. SHE) and low-energy region (0.1 eV ~ −0.05 eV *vs*. SHE) in this system. Thus, the photogenerated electrons at low-energy level would quickly relax to the VB bottom of α-Fe_2_O_3_, then to recombine with holes. Meanwhile, partial high-energy electrons would thermodynamically transfer to the CB of TiO_2_ due to their matchable energy band position and intimate interfacial contact, thus resulting in the improved fate of photogenerated electron-hole pairs. Taking pyridine denitrogenation as the model reaction, the TiO_2_/α-Fe_2_O_3_ photocatalysts have exhibited enhanced photocatalytic activity compared with pure TiO_2_ and α-Fe_2_O_3_. The higher photoactivity of TiO_2_/Fe_2_O_3_ can be attributed to the enhanced visible light absorption, efficient charge-carrier separation as well as the synergistic effect between TiO_2_ and α-Fe_2_O_3_. To the best of our knowledge, this work represents the first example to use the TiO_2_/α-Fe_2_O_3_ semiconductor composite photocatalyst for photocatalytic denitrification of NCCs. It is expected that our work could offer new inroads into explore heterojunction photocatalysts for photocatalytic denitrification of gasoline fuel.

## Methods

### Reagents and chemicals

All reagents and solvents were used as received from commercial suppliers without further purification. Tetrabutoxytitanium was supplied by Aladdin Reagent Co., Ltd. (Shanghai, China). Iron(III) nitrate nonahydrate (Fe(NO_3_)_3_·9H_2_O), ethylene glycol, pyridine, octane were supplied by Sinopharm Chemical Reagent Co., Ltd. (Shanghai, China).

### Synthesis of TiO_2_ sample

TiO_2_ were prepared using the method previously reported by Peng et.^28^. 3 mL of tetrabutoxytitanium was added to 30 mL ethylene glycol in a rockered flask. The solution was treated at 180 °C for 2 h under continuous magnetic stirring, the white slurry was formed. Then the solution was cooled to room temperature naturally. The final white solid products were centrifuged and washed with ethanol several times to ensure total removal of the excess ethanol and the dried at room temperature. The assynthesized white solid products were titanium glycolate. Finally, the titanium glycolate precursor was calcined at 450 °C for 2 h to form TiO_2_.

### Synthesis of TiO_2_/Fe_2_O_3_ samples

The TiO_2_/Fe_2_O_3_ composites were synthesized by wet impregnation, drying, ethanol washing, and calcination process. Typically, 30 mL of 0.3 M (0.6 M or 0.9 M) Fe(NO_3_)_3_·9H_2_O in ethanol (EtOH) was added to 1.5 g of TiO_2_ powder, stirring for 30 min at room temperature and then sonicating for 30 min. After that, the suspension was evaporated at 50 °C to obtain solid sample. Then the sample was calcined at 300 °C for 10 min. Next, the sample was washed by ethanol thoroughly. Finally, the sample was once again calcined at 300 °C for 6 h. The loading of α-Fe_2_O_3_ in the composites was about 30, 50, and 70 wt% for 0.3, 0.6, and 0.9 M Fe(NO_3_)_3_·9H_2_O, designated as sample TiO_2_/Fe_2_O_3_-3, TiO_2_/Fe_2_O_3_-5, and TiO_2_/Fe_2_O_3_-7, respectively.

### Characterization of materials

XRD patterns were carried on a Bruker D8 Advance X-ray diffrractometer operated at 40 kV and 40 mA with Ni-filtered Cu Kα irradiation (λ = 0.15406 nm). The data were recorded in the 2θ range of 10–80°. The Brunauer-Emmett-Teller (BET) surface area was measured with an ASAP2020M apparatus (Micromeritics Instrument Corp., USA). Before the test, the samples were degassed in vacuum at 240 °C for 6 hours. The nitrogen adsorption and desorption isotherms were measured at 77 K. UV-vis diffuse reflectance spectra (UV-vis DRS) were obtained by a UV-vis spectrophotometer (Shimadzu UV-2700) with BaSO_4_ as a reflectance standard. X-ray photoelectron spectroscopy (XPS) measurements were conducted on a PHI Quantum 2000 XPS system equipped with a monochromatic Al Kα X-ray source to obtain the surface elemental composition of the sample. The concentration of Fe_2_O_3_ in the sample was detected by the Ultima2 ICP optical emission spectrometer. The magnetization curves were measured at room temperature under a varying magnetic field from −4 to + 4 kOe on a BHV-55 vibrating sample magnetometer (VSM). Temperature-programmed desorption (TPD) of ammonia was conducted in a flow apparatus on a Micrometrics 2910 Autochem analyzer. In a typical NH_3_-TPD experiment, about 0.2 g of the sample was loaded in U-shaped quartz cell above a small amount of quartz wool. Before the experiments, the samples were pretreated for 2 h at 400 °C in a flow of helium. NH_3_-TPD was carried out in helium flow after purging the sample at 50 °C during 60 min to decrease the amount of physisorbed ammonia. The temperature was increased with a rate of 10 °C/min up to 700 °C. The electrochemical measurements were performed in a conventional three electrode cell, Ag/AgCl electrode was used as the reference electrode and a Pt plate was used as the counter electrode. The photocurrent measurements were conducted with a BAS Epsilon workstation. The liquid chromatograph-mass spectrometer (HPLC-MS) methods for analyzing pyridine was performed using an Agilent 1200 series (Palo Alto, CA, USA) equipped with an Agilent Zorbax Eclipse XDB-C18 column (2.1 mm × 100 mm, 3.5 m). The column was maintained at 30 °C during the sample analysis. The measurement for pyridine was performed in an isocratic elution program with methanol/acetone = 70:30 (v/v) as mobile phase. Flow rate was kept at 0.2 mL/min, and the injection volume was10 μL.

### Evaluation of photocatalytic activity

Simulated NCCs-containing gasoline fuel of 100 μg/g was prepared by dissolving 70 mg of pyridine in 1.0 L of octane. The octane interaction with hydroxylated surfaces of TiO_2_/Fe_2_O_3_ has been shown to be very weak and nonspecific, while the interaction of polar compounds such as pyridine is expected to be stronger. Furthermore, considering that octane is the main ingredient of gasoline, which is low-cost and low toxicity. Therefore, we have chosen octane as the optimum reaction solvent in our reaction system. The photocatalytic denitrification of pyridine was carried out at 30 °C in a 100 mL quartz reactor containing 40 mg of TiO_2_/Fe_2_O_3_ and 40 mL of pyridine/octane solution (100 μg/g). The suspension was stirred in the dark for 1 h to ensure the establishment of adsorption-desorption equilibrium, the suspensions were irradiated by a 300 W Xe lamp (PLS-SXE 300, Beijing Perfectlight Co. Ltd) with a UV-CUT filter to cut off light of wavelength < 420 nm. During illumination, 2 mL of suspension was taken from the reactor at a scheduled interval and centrifuged to separate the photocatalyst. The pyridine content in the supernatant solution was determined colorimetrically at 251 nm using a Cary 50 UV-vis spectrophotometer (Varian Co.). In order to quantitatively understand the reaction kinetics of the pyridine photocatalytic denitrification in our experiments, we applied the pseudo-first order model as expressed by Eq. (1), which is generally used for photocatalytic degradation process if the initial concentration of pollutant is low:

1 $$\mathrm{ln}({{\rm{C}}}_{{\rm{0}}}{/C}_{{\rm{t}}})={\rm{kt}}$$

where C_0_ and C_t_ are the concentrations of the pollutants in solution at time 0 and t, respectively, and k is the pseudo-first order rate constant.

## Electronic supplementary material

Supporting Information
