# Supplementary material for: Enhanced Photocatalytic Fuel Denitrification over TiO2/α-Fe2O3 Nanocomposites under Visible Light Irradiation
Source: Sci Rep. 2017 Aug 10;7:7858. doi: 10.1038/s41598-017-08439-3 (PMC5552819; doi:10.1038/s41598-017-08439-3)
Supplement: Supplementary file 1 — Supporting Information [file 41598_2017_8439_MOESM1_ESM.doc]

**Supplementary information**

**Enhanced Photocatalytic Fuel Denitrification over TiO_2_/α-Fe_2_O_3_ Nanocomposites under Visible Light Irradiation**

Renkun Huang ^1^, Ruowen Liang ^1^, Haimei Fan ^1^, Shaoming Ying^1^, Ling Wu ^2^,

Xuxu Wang ^2^ and Guiyang Yan^1,*^

^1^ Department of chemistry, Fujian province university key laboratory of green energy and environment catalysis, Ningde Normal University, Ningde 352100, P. R. China

^2^ State key laboratory of photocatalysis on energy and environment, Fuzhou University, Fuzhou 350002, P. R. China

* Corresponding author: Prof. Guiyang Yan, E-mail: ygyfjnu@163.com


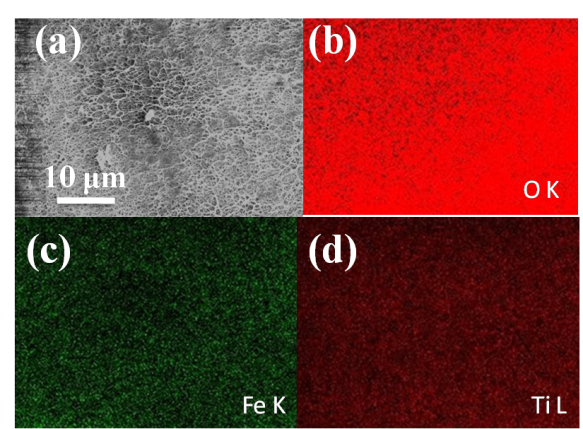


**Figure S1.** The mapping images of TiO_2_/Fe_2_O_3_-5.


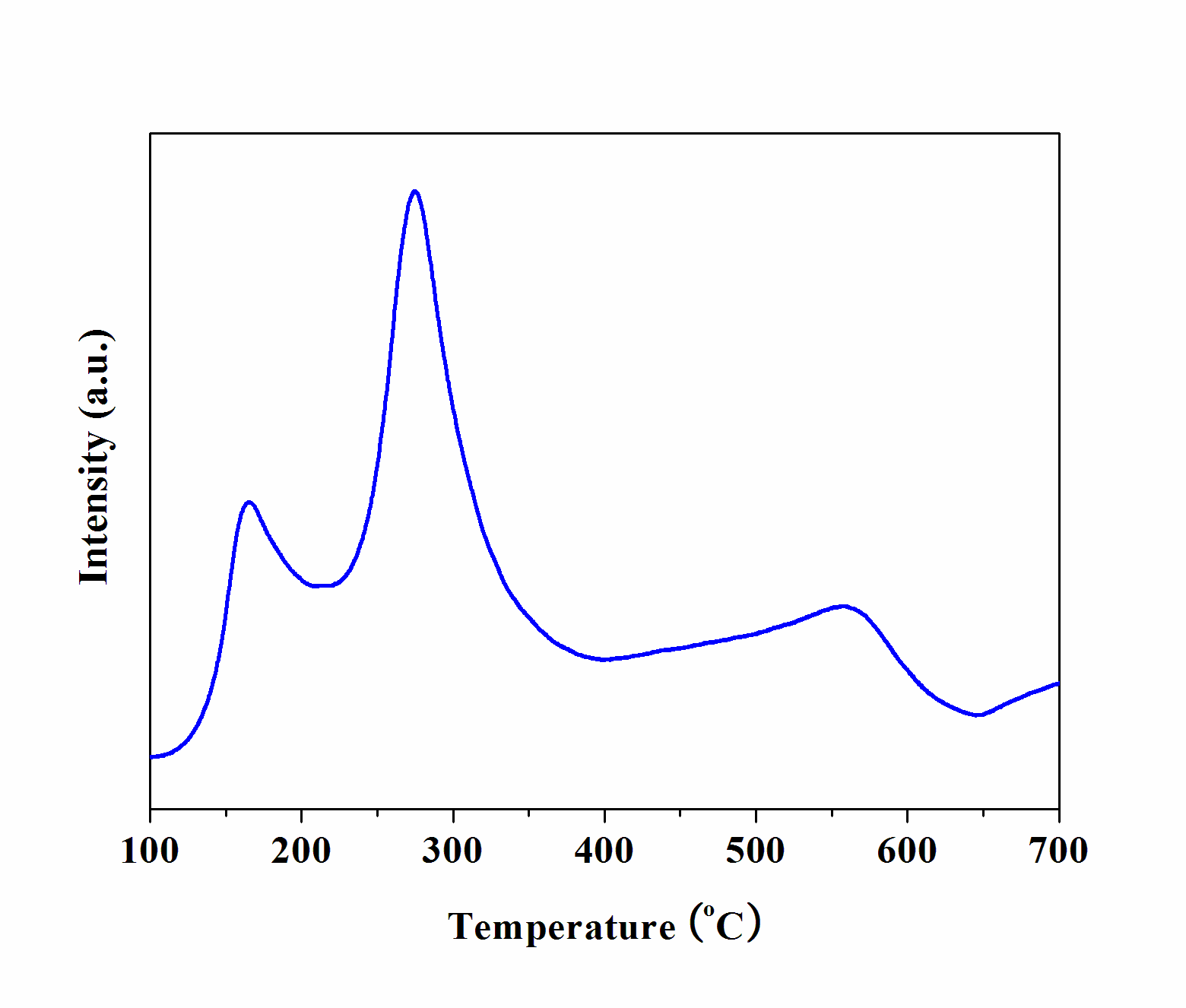


**Figure S2.** NH_3_-TPD profiles of TiO_2_/Fe_2_O_3_-5.


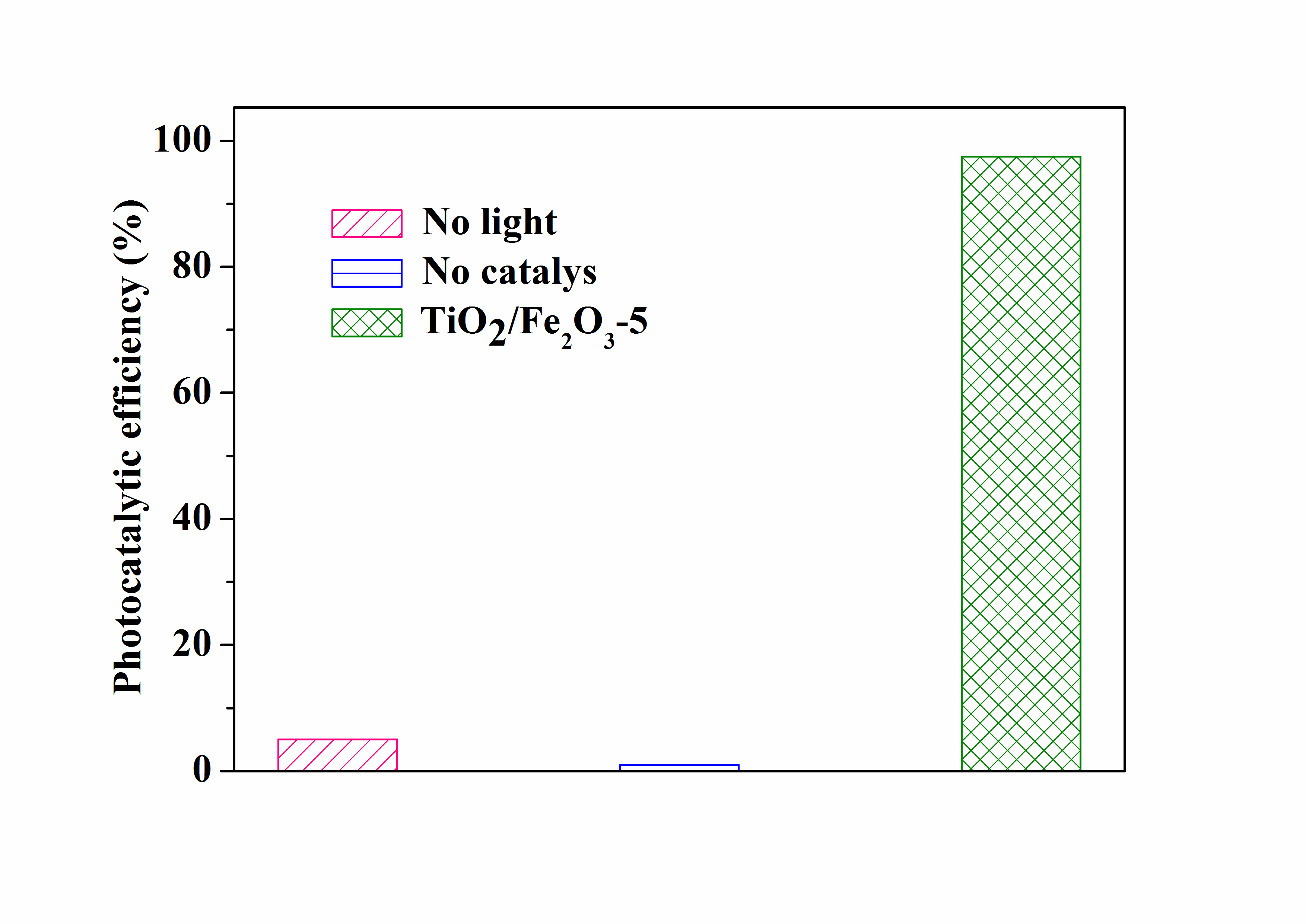


**Figure S3.** Control experiments of photocatalytic denitrogenation of pyridine under different conditions. Reaction conditions: 40 mg of photocatalyst, 40 mL of 100 μg/g pyridine.


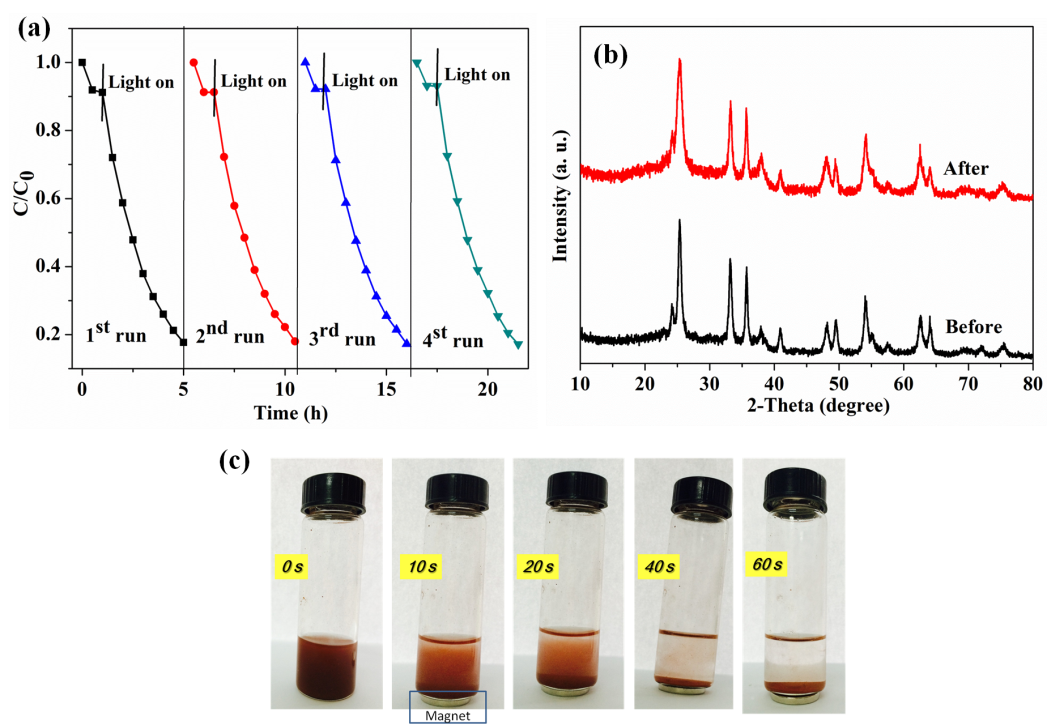


**Figure S4.** (a) Reusability of 5-TiO_2_/Fe_2_O_3_ for photocatalytic denitrogenation of pyridine; (b) XRD patterns of 5-TiO_2_/Fe_2_O_3_ before and after the catalytic reaction; (c) separation of the sample from solution under an external magnetic field.


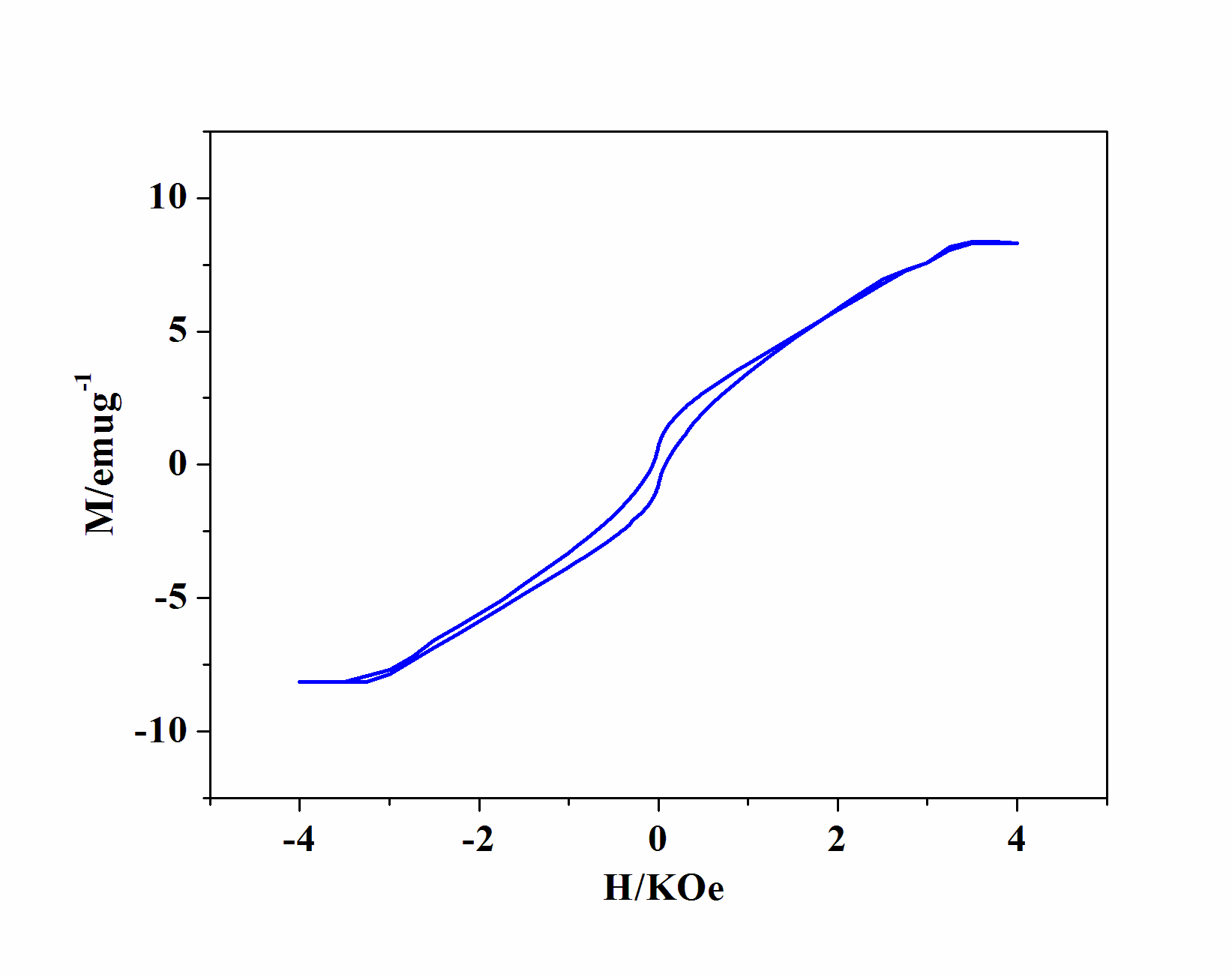


**Figure S5.** Room-temperature magnetization curves of the TiO_2_/Fe_2_O_3_-5.


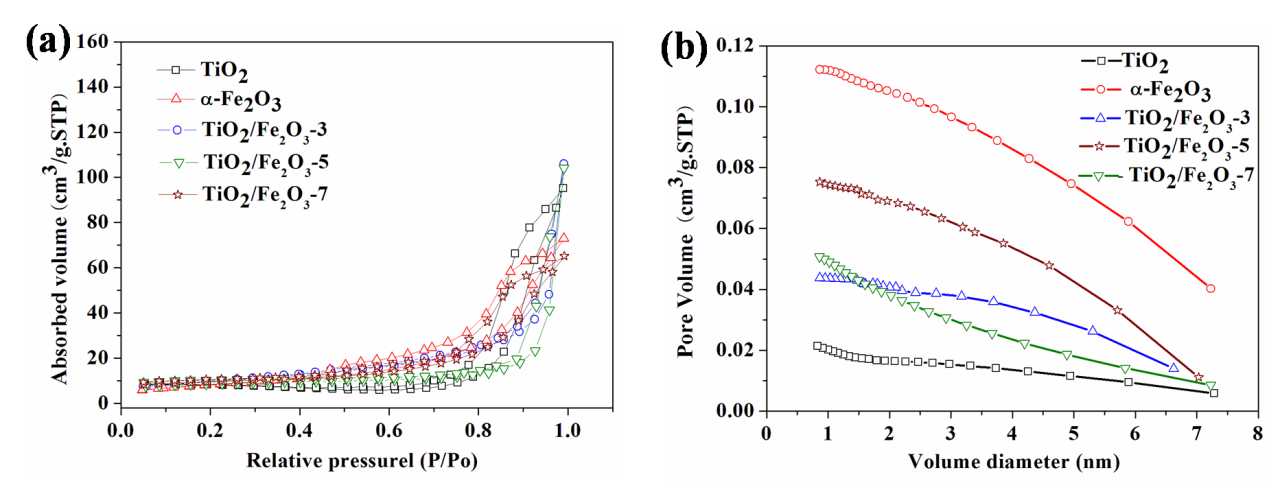


**Figure S6.** (a) BET adsorption-desorption isotherm and (b) pore size distribution of TiO_2_, α-Fe_2_O_3_ and TiO_2_/Fe_2_O_3_ composites.


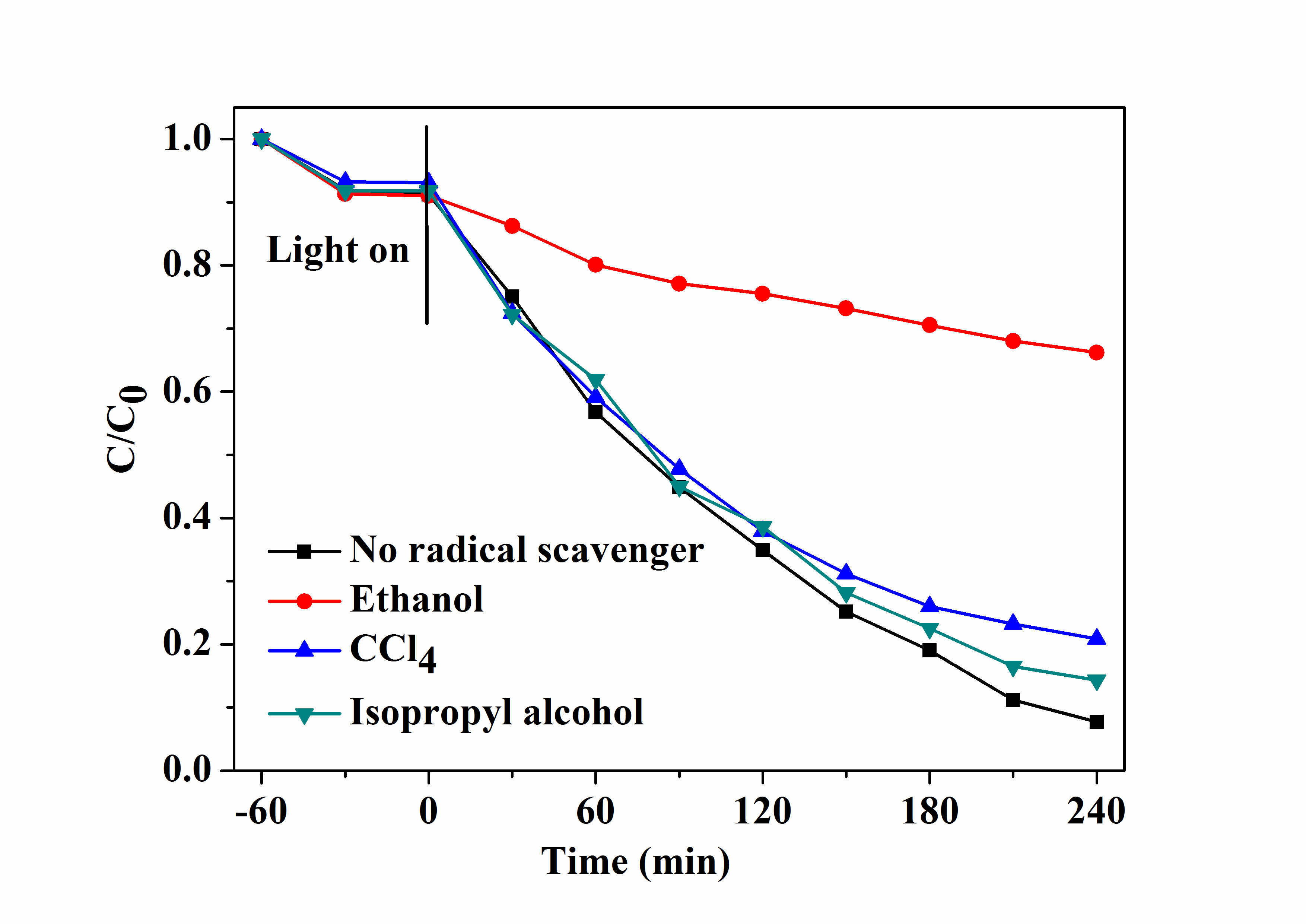


**Figure S7.** The photocatalytic denitrogenation of pyridine over 5-TiO_2_/Fe_2_O_3_ under visible light irradiation the presence of radical scavengers (0.1 mmol).

**
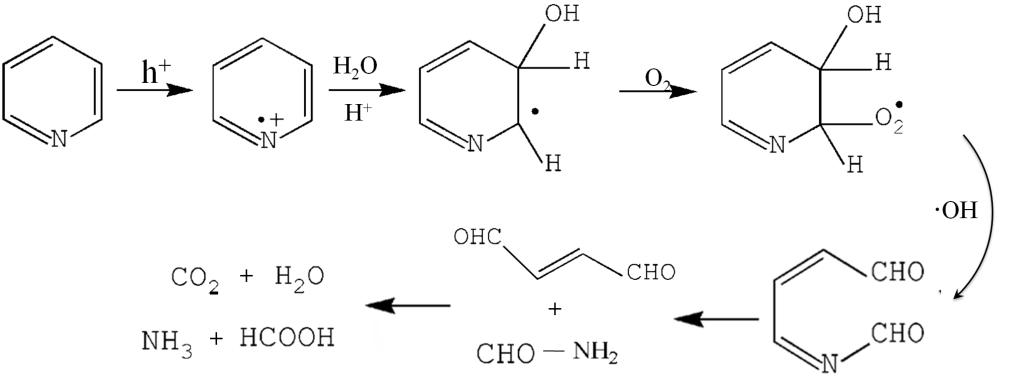
**

**Figure S8.** Possible denitrogenation pathway of pyridine.

**
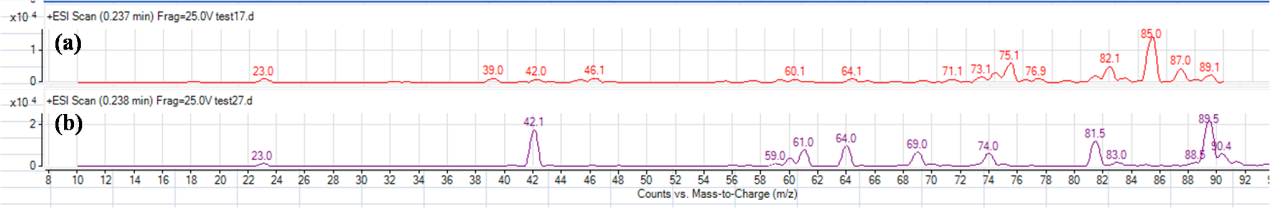
**

**Figure S9.** HPLC profiles of pyridine after different irradiation times: (a) 4 h and (b) 0 h.
